# Real-World Assessment of Direct Oral Factor Xa Inhibitors: Dosing Appropriateness, Drug Exposure, and Inter-Platform Comparability of Chromogenic Anti-Xa Assays

**DOI:** 10.3390/diagnostics16172725

**Published:** 2026-08-26

**Authors:** Yoonjung Kim, Sojin Lee, Yongjung Park, Kyung-A Lee

**Affiliations:** 1 Department of Laboratory Medicine, Gangnam Severance Hospital, Yonsei University College of Medicine, Seoul 06273, Republic of Korea; wsyj1101@yuhs.ac (Y.K.); 2sojin1009@yuhs.ac (S.L.); 2 Department of Laboratory Medicine, Yongin Severance Hospital, Yonsei University College of Medicine, Yongin 16995, Republic of Korea

**Keywords:** anticoagulants, atrial fibrillation, blood coagulation tests, drug monitoring, factor Xa inhibitors

## Abstract

**Background/Objectives:** Direct oral factor Xa inhibitors (DOACs) show substantial interindividual exposure variability in real-world practice, yet interpretation of plasma drug concentrations remains challenging because routine monitoring is not standardized. This study evaluated dosing appropriateness, plasma drug exposure measured by chromogenic anti-factor Xa assays, inter-platform comparability, and associations with routine coagulation tests. **Methods:** This single-center retrospective study included 392 plasma samples from 292 patients with atrial fibrillation treated with apixaban (*n* = 207), edoxaban (*n* = 147), or rivaroxaban (*n* = 38). Post-dose outpatient spot samples were analyzed using Sysmex CS-5100 and CN-6000 analyzers with two chromogenic anti-factor Xa assay systems (BIOPHEN™ DiXaI and BIOPHEN™ Heparin LRT) and drug-specific calibrators. Correlations between routine assays (prothrombin time [PT]/international normalized ratio [INR], activated partial thromboplastin time [aPTT]) and DOAC concentrations, as well as inter-platform agreement, were assessed using Spearman’s rank correlation and Passing-Bablok regression. Dosing appropriateness was determined according to Food and Drug Administration-approved labeling. **Results:** Overall dosing appropriateness was 69.2% (edoxaban 73.3%, apixaban 70.5%, rivaroxaban 45.5%), whereas inappropriate underdosing was the predominant prescribing pattern (26.9%), particularly among patients without formal dose-reduction criteria. PT/INR demonstrated the strongest correlation with edoxaban concentrations (rs = 0.83, *p <* 0.001). Inter-analyzer correlation between the CS-5100 and CN-6000 was excellent (r = 0.885–0.996), with LRT reagents providing highly consistent results across platforms. **Conclusions:** Real-world outpatient DOAC concentrations demonstrated substantial variability and frequent off-label underdosing. Chromogenic anti-factor Xa assays showed strong inter-platform agreement, particularly with LRT reagents, supporting their analytical reliability for laboratory assessment of DOAC exposure. Because exact dosing times were unavailable, these concentrations should be interpreted as heterogeneous spot samples rather than standardized pharmacokinetic reference intervals; these findings may nonetheless support individualized interpretation of outpatient spot sample DOAC concentrations in selected clinical settings.

## 1. Introduction

Direct oral anticoagulants (DOACs), also known as non-vitamin K antagonist oral anticoagulants, are widely used for stroke and systemic embolism prevention in patients with non-valvular atrial fibrillation (NVAF), perioperative venous thromboembolism prophylaxis after major orthopedic surgery, and the treatment and secondary prevention of deep vein thrombosis and pulmonary embolism [1,2]. Warfarin therapy requires frequent laboratory monitoring and dose adjustment to maintain therapeutic anticoagulation, whereas DOACs provide more predictable exposure–response relationships and generally do not require routine monitoring in most clinical settings [2,3].

Because a proportion of each agent is eliminated by the kidney, approximately 27% for apixaban, 33% for rivaroxaban, 50% for edoxaban, and 80% for dabigatran, plasma concentrations are particularly sensitive to renal function [4]. Consequently, dosing recommendations incorporate renal function and, depending on the agent, age, body weight, and concomitant medications, inappropriate dosing may increase thromboembolic risk when underdosed or bleeding risk when overdosed, underscoring the importance of confirming dose appropriateness in patients near dose-adjustment thresholds or with dynamic renal function. Contemporary atrial fibrillation guidance reinforces this concern, noting that inappropriate dose-reduction can increase thromboembolic risk without a corresponding reduction in bleeding risk and underscoring the importance of adhering to full DOAC dosage to avoid preventable thromboembolic events [5].

Although routine monitoring is generally not required, measurement of DOAC exposure can be clinically informative in selected scenarios, such as extremes of body weight, renal dysfunction, advanced age, suspected drug interactions, urgent surgery or invasive procedures, thrombosis occurring on therapy, and major bleeding events [3].

Plasma concentrations of factor Xa inhibitors can be measured using liquid chromatography–tandem mass spectrometry (LC–MS/MS) or chromogenic anti-factor Xa assays [6,7,8]. Although LC–MS/MS is considered the reference method because of its analytical specificity and accuracy, it is limited for routine use by costs, technical requirements, and turnaround time [9,10]. Conversely, automated chromogenic anti-factor Xa assays offer practical advantages, including rapid turnaround time and scalability, making them suitable for therapeutic drug monitoring and time-sensitive decision-making in routine clinical laboratories. However, quantitative results may vary by reagents, calibrators, and analytical platforms, highlighting the importance of method validation and inter-platform comparability before the application of concentration-guided management [11].

Despite growing interest in individualized DOAC management, real-world data on plasma DOAC concentrations across dosing regimens and stratified by renal function remain limited, and therapeutic reference ranges are not uniformly established. Consequently, interpretation of measured DOAC levels remains challenging and method-dependent. Given the persistent risks of thromboembolic and bleeding events in contemporary AF cohorts (1–3% per year) [12,13,14], drug exposure must be contextualized within real-world clinical practice. Few studies have jointly evaluated DOAC dosing appropriateness, real-world drug exposure, and inter-platform/inter-reagent analytical comparability within a single cohort.

In this study, we analyzed plasma concentrations of apixaban, edoxaban, and rivaroxaban measured using chromogenic anti-factor Xa assays in patients with NVAF treated at a tertiary hospital in South Korea. We assessed dose appropriateness according to product labeling, characterized variability in drug exposure across DOAC types and dosing regimens, and evaluated analytical performance, including correlations with routine coagulation markers. By integrating clinical dosing patterns, laboratory measurements, and renal function data, this study aims to provide practical insights to support individualized anticoagulation management and inform quality improvement initiatives in prescribing DOAC.

## 2. Materials and Methods

### 2.1. Study Design and Population

In this single-center retrospective study, adult patients with NVAF or atrial flutter treated with apixaban, edoxaban, or rivaroxaban for more than 3 months were eligible for enrollment. Blood samples were collected from patients treated at our center between December 2020 and July 2023.

Patients aged <18 years, those with DOAC concentrations below the lower limit of quantification (LoQ), and those with incomplete clinical data were excluded. For each patient, the following data were recorded: DOAC agent, dose, and dosing frequency; demographic variables (age, sex, height, and weight); indication for anticoagulation; concomitant medications (antiplatelet agents, nonsteroidal anti-inflammatory drugs, and selective serotonin reuptake inhibitors); laboratory parameters (serum creatinine, prothrombin time [PT]/international normalized ratio [INR], and activated partial thromboplastin time [aPTT]); and clinical risk scores (CHA_2_DS_2_-VASc and HAS-BLED).

Renal function was assessed using estimated glomerular filtration rate (eGFR), calculated using the Modification of Diet in Renal Disease (MDRD) and the 2009 Chronic Kidney Disease Epidemiology Collaboration (CKD-EPI) creatinine equations [15]. For dose appropriateness classification, creatinine clearance (CrCl_CG) was calculated using the Cockcroft-Gault equation based on age, body weight, sex, and serum creatinine values, according to approved labeling criteria for direct oral anticoagulants.

### 2.2. DOAC Concentration Measurement

Blood samples were collected from outpatients during routine clinic visits when coagulation testing was ordered as part of standard care. Patients were instructed to take their regular medications, including DOACs, with a small amount of water on the morning of their clinic visit. Consequently, measured concentrations represent post-dose spot samples rather than standardized trough (pre-dose) levels. Sampling time was descriptively summarized using clock-time windows (<06, 06–10, 10–14, 14–18, 18–24). Blood samples were collected in 3.2% sodium citrate tubes. Plasma was separated by centrifugation within 1 h of collection and stored at −80 °C until analysis.

DOAC plasma concentrations were measured using two automated coagulation analyzers, the Sysmex CS-5100 and Sysmex CN-6000 (Sysmex Corporation, Kobe, Japan). For each analyzer, concentrations were assessed using two chromogenic anti-factor Xa assay systems. The BIOPHEN™ DiXaI kit (HYPHEN BioMed, Neuville-sur-Oise, France), a two-stage chromogenic assay specific for direct factor Xa inhibitors (rivaroxaban, apixaban, and edoxaban), was performed using drug-specific calibrators according to the manufacturer’s instructions. The BIOPHEN™ Heparin LRT (Liquid Reagent Technology) assay (HYPHEN BioMed, Neuville-sur-Oise, France), a liquid chromogenic anti-factor Xa assay applicable to direct Xa inhibitors, was performed using drug-specific calibrators. Consequently, four measurements were obtained for each plasma sample: CS-5100 (DiXaI), CS-5100 (LRT), CN-6000 (DiXaI), and CN-6000 (LRT). The LoQ for quantitative DOAC reporting was defined according to the manufacturer’s specifications for each analyzer and reagent combination. For analyses requiring quantitative concentration values, specimens with concentrations below the LoQ were excluded, as such measurements do not allow reliable quantitative interpretation. Routine coagulation testing (PT/INR, aPTT) was performed on the Sysmex CN-6000 analyzer using the manufacturer’s default analyzer settings, with Thromborel S (Siemens Healthineers, Erlangen, Germany; lot 572175) for PT/INR and ACTIN FSL (Siemens Healthineers, Erlangen, Germany; lot 575055) for aPTT. The laboratory reference ranges were 10.1–12.8 s (79.4–130.7%; INR 0.87–1.11) for PT and 22.4–33.2 s for aPTT, with coefficients of variation of 1.5–2.2% for PT and 3% for aPTT.

### 2.3. Assumption-Based Pharmacokinetic-Window Classification

Because the exact time of DOAC administration was not available in this outpatient dataset, the post-administration time was estimated by assuming a uniform morning dosing time and calculating the elapsed time to blood sampling. Samples were subsequently categorized into descriptive elapsed-time windows (1–4 h, 4–8 h, 8–12 h, >12 h) after the assumed dose, solely to visualize real-world spot-sampling patterns rather than to reproduce a protocolized peak–trough design. Details of the PK-window definition and supporting pharmacokinetic rationale are provided in the Supplementary Methods S1.

### 2.4. Classification of Dose Appropriateness

Dose appropriateness was determined by comparing the prescribed regimen with dose-reduction criteria based on Food and Drug Administration (FDA)-approved labeling. For apixaban, the standard dose was 5 mg twice daily and the reduced dose was 2.5 mg twice daily if ≥2 of the following were present: age ≥ 80 years, body weight ≤ 60 kg, or serum creatinine ≥ 1.5 mg/dL. For edoxaban, the standard dose was 60 mg once daily and the reduced dose was 30 mg once daily for CrCl_CG 15–50 mL/min or body weight ≤ 60 kg. For rivaroxaban, the standard dose was 20 mg once daily and the reduced dose was 15 mg once daily for CrCl_CG 15–50 mL/min for risk reduction of stroke in patients with NVAF. Patients were categorized as receiving a recommended dose or a reduced/non-recommended dose.

### 2.5. Post-Hoc and Exposure Outlier Analysis

Post-hoc descriptive analyses were performed to summarize dose modification patterns, clinical events, and analytical exposure outliers. Detailed post-hoc review procedures are described in the Supplementary Methods S2.

### 2.6. Statistical Analysis

Continuous variables are presented as mean ± standard deviation or median (interquartile range), as appropriate, and categorical variables as counts and percentages. Baseline characteristics were compared using the Kruskal–Wallis test, given the skewed distribution of most continuous variables, for continuous variables and the chi-square test or Fisher’s exact test for categorical variables. Method comparisons between analyzers and assay formats were performed using Passing–Bablok regression and Bland–Altman analysis. Central 95% concentration intervals for DOAC plasma concentrations were estimated using percentile-based methods (non-parametric estimation for apixaban and edoxaban, *n* > 120). Spearman’s rank correlation coefficient was used to assess associations between DOAC concentrations and continuous variables.

For patient-level analyses, including baseline characteristics and dose appropriateness, only the first available sample from each patient was used. Analytical method-comparison analyses retained all valid samples because measurements obtained using different analyzers or assay formats were paired within the same plasma specimen. Variables with missing data were excluded on a per-analysis basis using available-case data; variable-specific sample sizes are reported in the corresponding tables and Supplementary Materials.

A two-sided *p*-value < 0.05 was considered statistically significant. The Benjamini–Hochberg false-discovery-rate correction was applied to multiple correlation analyses, with q < 0.05 considered significant. All analyses were performed using R version 4.3.2 with the mcr, PMCMRplus, and boot packages.

## 3. Results

### 3.1. Study Population

**Figure 1:**
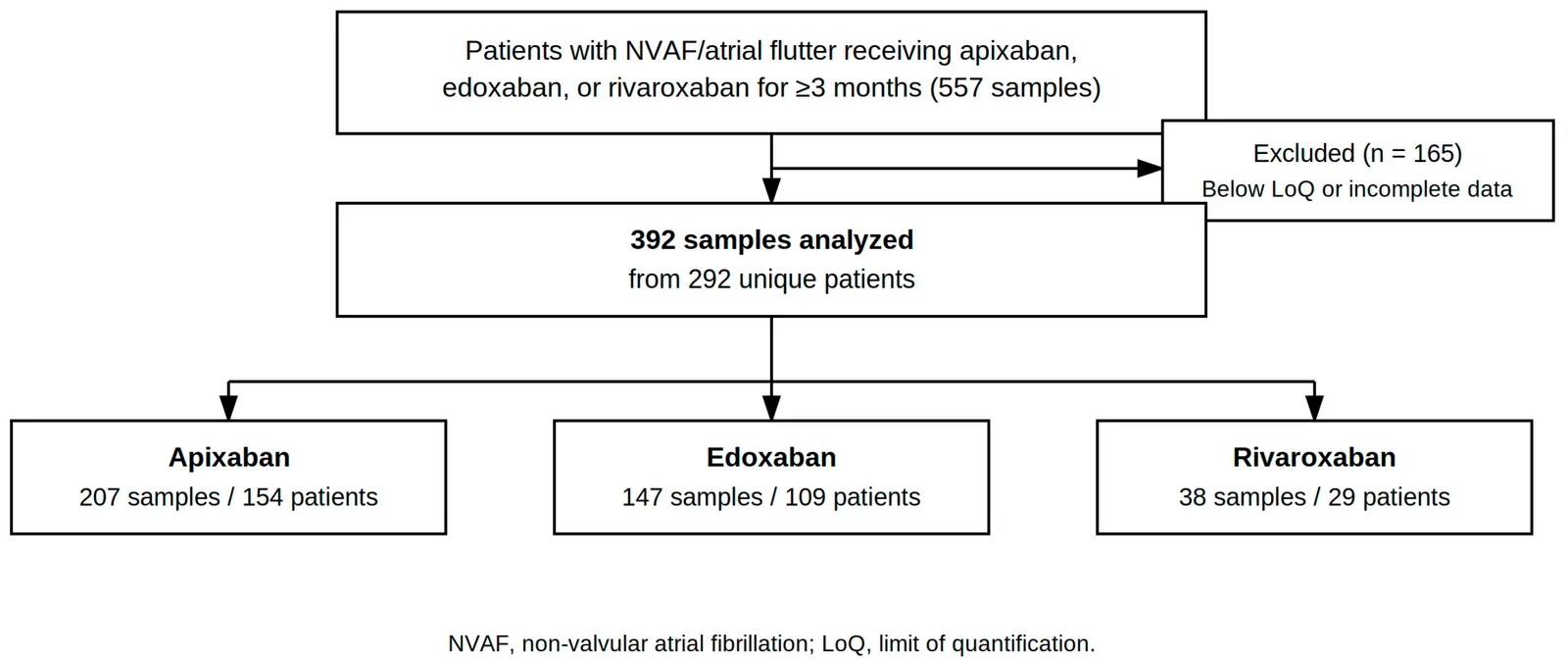
Study flow diagram. A total of 557 plasma samples were collected from outpatients with non-valvular atrial fibrillation (NVAF) or atrial flutter treated with apixaban, edoxaban, or rivaroxaban for ≥3 months. After excluding 165 samples that were below the analytical limit of quantification (LoQ) or had incomplete clinical data, 392 samples from 292 unique patients (identified by electronic medical record registration number) were included in the final analysis: apixaban (207 samples/154 patients), edoxaban (147 samples/109 patients), and rivaroxaban (38 samples/29 patients). NVAF, non-valvular atrial fibrillation; LoQ, limit of quantification.

A total of 557 plasma samples were collected from patients receiving DOAC therapy, including 239 apixaban, 264 edoxaban, and 54 rivaroxaban samples. After excluding samples below the analytical limit of quantification or with incomplete clinical data (Figure 1), 392 samples from 292 unique patients were included in the final analysis. These comprised 207 samples from 154 patients receiving apixaban, 147 samples from 109 patients receiving edoxaban, and 38 samples from 29 patients receiving rivaroxaban. Of the 292 patients, 217 contributed a single sample, whereas 75 (25%) contributed two to five samples collected during separate outpatient visits.

Baseline characteristics are summarized in Table 1. The median age was 73 years in all three DOAC groups and did not differ significantly among patients receiving apixaban, edoxaban, or rivaroxaban (*p* = 0.670). Sex distribution, body weight, height, and body mass index were also comparable across the groups. Renal function did not differ significantly by DOAC type. Median CKD-EPI eGFR values were 72.0 mL/min/1.73 m^2^ for apixaban, 72.0 mL/min/1.73 m^2^ for edoxaban, and 83.0 mL/min/1.73 m^2^ for rivaroxaban (*p* = 0.074). Median Cockcroft–Gault creatinine clearance values were 59.2, 56.8, and 68.2 mL/min, respectively (*p* = 0.580). Although the *p*-values differed between CKD-EPI eGFR and Cockcroft–Gault creatinine clearance, neither comparison reached statistical significance. Serum creatinine concentrations were likewise comparable among the groups (*p* = 0.188). Thromboembolic and bleeding risk scores did not differ significantly according to DOAC type. Median CHA_2_DS_2_-VASc scores were 3.0 for apixaban, 3.0 for edoxaban, and 4.0 for rivaroxaban (*p* = 0.745), while the median HAS-BLED score was 1.0 in all three groups (*p* = 0.240). Concomitant antiplatelet use differed among the groups (*p* = 0.041), occurring in 20.8% of patients receiving apixaban, 32.1% receiving edoxaban, and 13.8% receiving rivaroxaban. Concomitant use of nonsteroidal anti-inflammatory drugs and selective serotonin reuptake inhibitors was uncommon and did not differ significantly among the groups.

Baseline characteristics are reported at the patient level. Each patient contributes one measurement, defined as the first available sample per unique electronic medical record (EMR) registration number. Among the 292 unique patients, 75 (25.7%) contributed two to five samples collected during separate outpatient visits. Baseline characteristics were therefore analyzed at the patient level using only the first available sample for each unique EMR registration number, and repeated samples were excluded from Table 1 to preserve statistical independence.

### 3.2. Clock-Time Sampling Patterns and Assumption-Based PK Window

Supplementary Table S1 summarizes outpatient clock-time windows (06–10/10–14/14–18) reflecting the clinical workflow, whereas Figure S1 summarizes assumed elapsed-time windows (1–4 h, 4–8 h, 8–12 h, >12 h) derived from time since last dose under a fixed dosing assumption (e.g., 07:00). In our cohorts, sampling was performed in the morning and midday windows, with wide and overlapping concentration ranges within each clock-time stratum.

Because the earliest elapsed-time window (1–4 h) under a 07:00 AM assumption (08:00–11:00) spanned more than one clock window, clock-time categories did not map uniquely to the assumed elapsed-time windows, which explained the discordance between values in Supplementary Table S1 and Figure S1.

### 3.3. Dose Appropriateness

At the patient level (first available sample per unique patient with complete dose-reduction criteria; *n* = 234), 162 patients (69.2%) received appropriate DOAC dosing according to FDA-approved labeling, whereas 72 (30.8%) received an inappropriate dose (Table 2). Dose appropriateness varied significantly by DOAC type (*p* = 0.036), with edoxaban demonstrating the highest rate (73.3%), followed by apixaban (70.5%) and rivaroxaban (45.5%). Inappropriate underdosing occurred in 63 patients (26.9% of the cohort; *p* = 0.197 between DOACs: rivaroxaban 40.9%, apixaban 27.9%, edoxaban 22.2%). Inappropriate overdosing was less common, affecting nine patients (3.8% of the cohort; *p* = 0.025: rivaroxaban 13.6%, edoxaban 4.4%, apixaban 1.6%).

**Figure 2:**
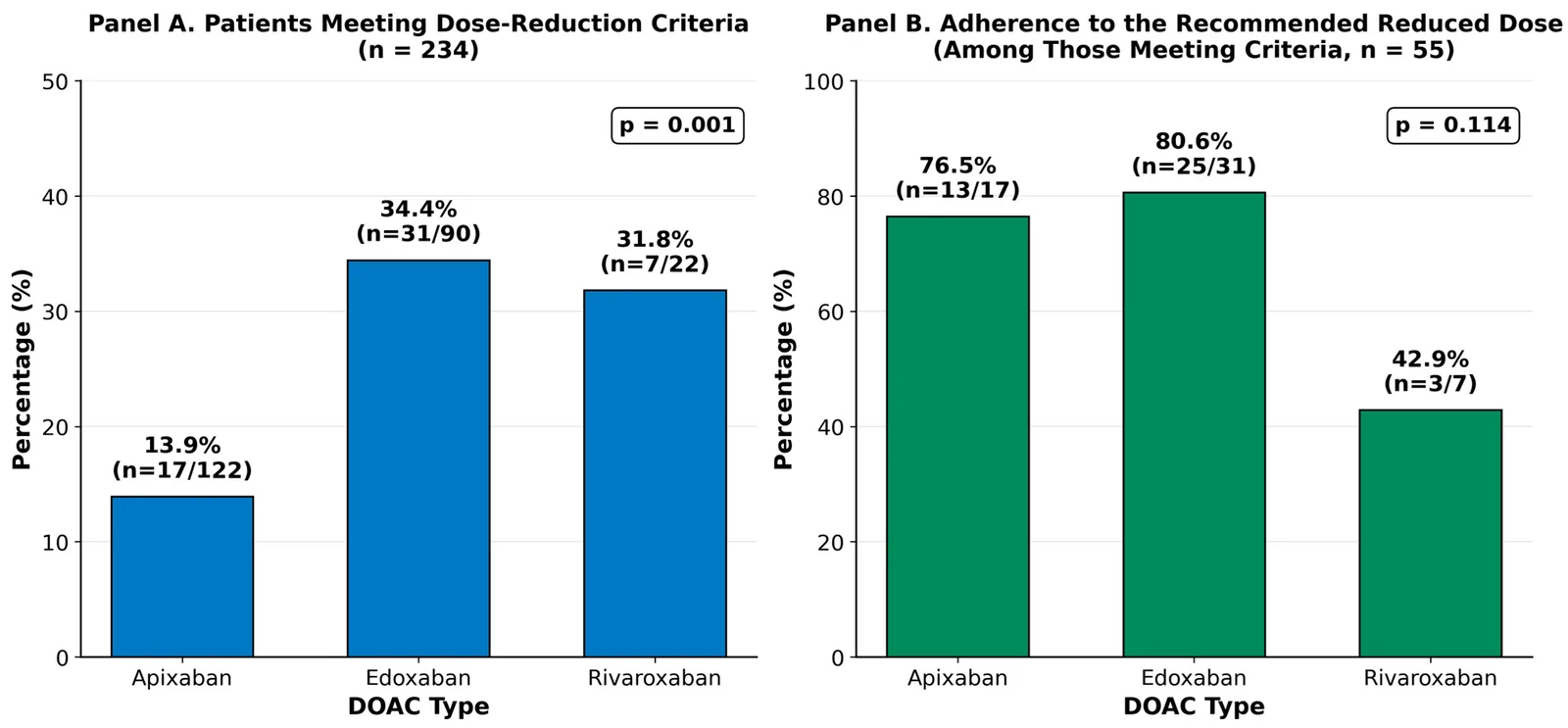
Dose-reduction criteria and adherence by DOAC type. (**A**) Proportion of patients meeting the Food and Drug Administration (FDA)-approved dose-reduction criteria according to DOAC type. Edoxaban had the highest proportion of patients meeting the criteria (34.4%, 31/90), followed by rivaroxaban (31.8%, 7/22) and apixaban (13.9%, 17/122; overall *p* = 0.001). (**B**) Adherence to the recommended reduced dose among patients meeting dose-reduction criteria. Edoxaban showed the highest adherence rate (80.6%, 25/31), followed by apixaban (76.5%, 13/17) and rivaroxaban (42.9%, 3/7; overall *p* = 0.114). DOAC, direct oral anticoagulant; FDA, Food and Drug Administration.

The proportion of patients meeting dose-reduction criteria also differed significantly by DOAC type (55/234, 23.5% overall; *p* = 0.001), being highest for edoxaban (31/90, 34.4%), followed by rivaroxaban (7/22, 31.8%) and apixaban (17/122, 13.9%). Among patients meeting dose-reduction criteria, adherence to the recommended reduced dose also varied by DOAC (*p* = 0.114): edoxaban had the highest adherence (25/31, 80.6%), followed by apixaban (13/17, 76.5%) and rivaroxaban (3/7, 42.9%) (Table 2, Figure 2).

Among the 55 patients who met dose-reduction criteria, 41 (74.5%) received the recommended reduced dose and were classified as appropriately dosed, while the remaining 14 (25.5%) received a dose other than the recommended reduced dose and were classified as inappropriately dosed (apixaban 4/17, edoxaban 6/31, rivaroxaban 4/7). Conversely, among the 179 patients who did not meet dose-reduction criteria, 121 (67.6%) appropriately received a standard (non-reduced) dose, whereas 58 (32.4%) received an inappropriately reduced or subtherapeutic dose despite not meeting reduction criteria (apixaban 32/105, edoxaban 18/59, rivaroxaban 8/15).

### 3.4. Correlation Between DOAC Concentrations and Clinical Parameters

**Figure 3:**
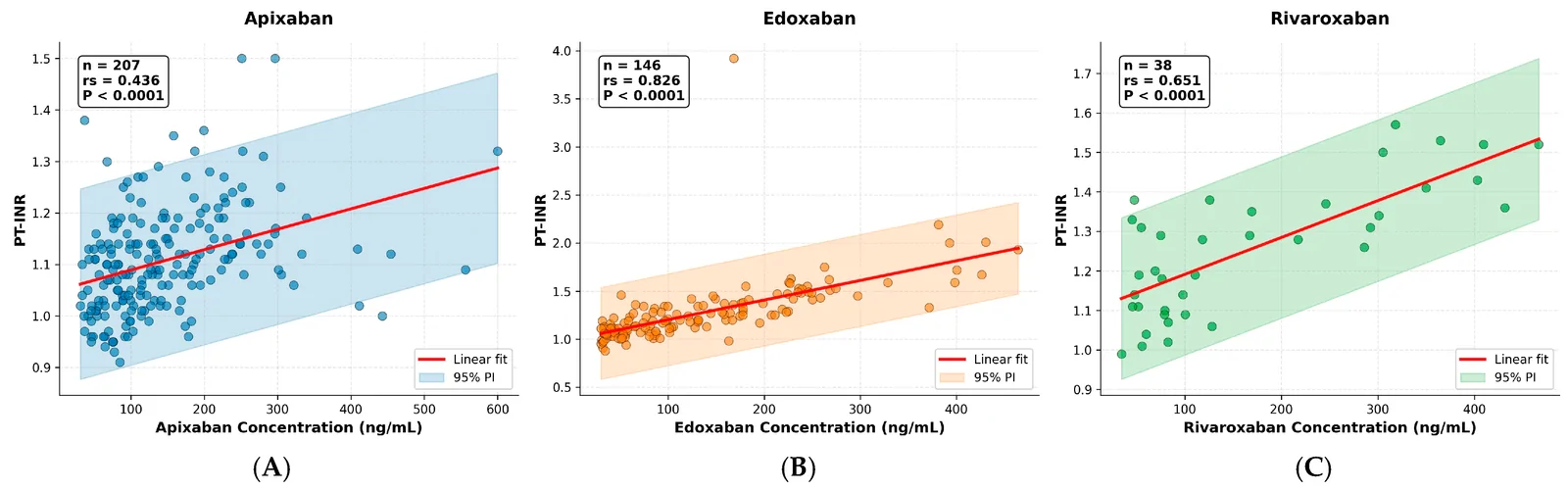
Correlation between DOAC concentrations and PT/INR. Scatter plots showing the relationship between DOAC plasma concentrations (measured by CN-6000 LRT) and PT/INR (with 95% prediction interval) for (**A**) apixaban (*n* = 207), (**B**) edoxaban (*n* = 146), and (**C**) rivaroxaban (*n* = 38). Red solid lines represent linear regression fits. Statistical significance was assessed using Spearman’s rank correlation coefficient (rs). All correlations were significant (*p* < 0.0001). DOAC, direct oral anticoagulant; PT, prothrombin time; INR, international normalized ratio. Red solid lines represent linear regression fits, shown to illustrate the visual trend; statistical inference was based on Spearman’s rank correlation coefficient (rs), which does not assume linearity.

Spearman correlation analysis revealed strong positive associations between DOAC plasma concentrations (CN-6000 LRT) and coagulation parameters (Supplementary Table S2). PT/INR demonstrated moderate correlations across all three DOACs, with the strongest correlation observed for edoxaban (Spearman’s rank correlation coefficient, rs = 0.83, *p* < 0.001), followed by rivaroxaban (rs = 0.65) and apixaban (rs = 0.44) (Figure 3). PT in seconds showed comparable correlations (rs = 0.45, 0.82, and 0.65, respectively; all *p* < 0.001). Edoxaban exhibited the strongest correlation with PT measurements. aPTT correlations were significant but more variable: apixaban rs = 0.15 (*p* = 0.037), edoxaban rs = 0.69 (*p* < 0.001), and rivaroxaban rs = 0.39 (*p* = 0.035).

Among demographic factors, age correlated weakly with apixaban concentration (rs = 0.14, *p* = 0.039), whereas body weight showed a weak positive correlation with edoxaban (rs = 0.20, *p* = 0.027). Other demographic and renal function variables showed no clinically meaningful correlations with DOAC concentrations.

### 3.5. Method Comparison Across DOAC Assays

Passing–Bablok regression demonstrated excellent agreement between CS-5100 and CN-6000 analyzers for all three DOACs (Table 3). Using DiXaI reagents, correlation coefficients were r = 0.885 for apixaban (*n* = 207 samples/154 patients), r = 0.990 for edoxaban (*n* = 146 samples; 1 sample excluded owing to an invalid CN-6000 reading on repeat testing), and r = 0.918 for rivaroxaban (*n* = 38 samples/29 patients). Liquid reagent technology (LRT) showed consistently higher correlations (r = 0.993–0.996 across all DOACs). Most, but not all, slope 95% confidence intervals (CIs) included 1.0 and most intercept 95% CIs included or approached 0. Specific comparisons showed statistically significant proportional or constant bias, notably the edoxaban LRT inter-analyzer comparison (slope 1.037, 95% CI 1.014–1.059; intercept −4.212, 95% CI −6.956 to −1.822), several within-analyzer DiXaI-vs.-LRT comparisons, and the rivaroxaban CN-6000 DiXaI-vs.-LRT comparison, indicating that inter-platform and inter-reagent agreement, although generally strong, does not by itself establish clinical interchangeability. Predefined acceptance criteria (mean bias within ±10%; ≥95% of paired differences within ±1.96 SD limits of agreement) were met for the primary CN-6000 LRT comparisons.

Bland–Altman analysis revealed minimal systematic bias among analyzers: apixaban −1.86 ng/mL, edoxaban −6.86 ng/mL, rivaroxaban −10.99 ng/mL (Supplementary Figure S2). Within-instrument comparisons between DiXaI and LRT reagents showed strong correlations (r = 0.855–0.996) (Supplementary Table S3), and LRT versus DiXaI reagent Bland–Altman analysis demonstrated 94.7–99.0% of measurements within limits of agreement (Supplementary Figure S3). These findings are consistent with limited platform-related differences under the tested conditions, though interchangeability claims require multicenter validation.

### 3.6. Observed Plasma Concentration Distributions of Direct Oral Factor Xa Inhibitors

The observed 95% concentration intervals in outpatient spot samples are summarized in Table 4. These values represent descriptive, real-world distributions derived from heterogeneous post-dose spot samples and should not be interpreted as validated reference or therapeutic intervals. All DOACs exhibited non-normal distributions (Anderson-Darling test, *p* < 0.05). Lower expected reference limits (2.5th percentile) remained consistent across all methods and DOACs. Upper expected reference limits (97.5th percentile) showed greater variability.

For rivaroxaban, the DiXaI method on the CS-5100 analyzer yielded a higher and more variable upper limit (750.9 ng/mL; 90% CI, 376.8–898.6) than LRT-based methods and the CN-6000 platform (450.4–458.6 ng/mL), likely reflecting outlier influence in the smaller cohort. In contrast, LRT-based methods produced narrower and more consistent real-world concentration distributions across both analyzers.

### 3.7. Post-Hoc Dosing Trajectories and Clinical Outcomes

To provide contextual, hypothesis-generating observations, we performed a brief post-hoc review of dosing trajectories among inappropriately dosed patients. This review was not based on a prespecified longitudinal follow-up protocol; follow-up duration varied across patients according to routine clinic visit patterns, and events were ascertained from available electronic medical records without formal adjudication. These observations should therefore be interpreted descriptively and are not intended to support an association between dosing appropriateness, measured concentration, and clinical outcomes.

Among the nine patients with inappropriate overdosing, a minority (*n* = 3) subsequently transitioned to appropriate dosing through dose-reduction or agent switching, while most (*n* = 5) remained inappropriately dosed; one patient was lost to follow-up.

Among the 63 patients with inappropriate underdosing, a minority (*n* = 20) transitioned to guideline-appropriate regimens through dose escalation or agent switching, while most (*n* = 28) remained inappropriately underdosed; 11 were lost to follow-up, and 4 died of causes unrelated to anticoagulation or thromboembolic disease during the observation period.

One fatal thromboembolic event was noted in the underdosing group, occurring in a patient with a high thromboembolic risk (CHA_2_DS_2_-VASc score 6) treated with low-dose edoxaban, who developed critical limb ischemia approximately two years after the index measurement. Given the prolonged interval and multiple comorbidities, no causal relationship with underdosing can be inferred from this single case. No major bleeding events were noted in this subgroup during the observation period.

Eighteen concentration outliers were identified, all confined to the upper tail of the distribution; these occurred across both appropriately and inappropriately dosed patients and were not associated with severe renal impairment. No thromboembolic or bleeding events were noted among these patients during the observation period.

## 4. Discussion

This study presents a real-world evaluation of DOAC dosing appropriateness, outpatient spot sample drug exposure, and analytical comparability of chromogenic anti-factor Xa assays across automated coagulation platforms. To our knowledge, few real-world studies have simultaneously evaluated dosing appropriateness, outpatient spot concentrations, and inter-platform agreement of chromogenic anti-factor Xa assays using multiple reagent systems in Asian patients receiving direct factor Xa inhibitors. Four principal findings emerged. First, off-label DOAC dosing was common, with 30.8% of patients receiving inappropriate doses and underdosing accounting for most cases (26.9%). Second, outpatient spot sample concentrations showed wide variability, highlighting the difficulty of interpreting DOAC levels without precise dosing-time information. Third, routine coagulation assays, particularly PT/INR, showed the strongest association with edoxaban concentrations (rs = 0.83), but remained insufficient as quantitative monitoring tools. Fourth, CS-5100 and CN-6000 analyzers demonstrated excellent inter-platform comparability, particularly with LRT reagents (r = 0.993–0.996).

Dose appropriateness varied significantly by DOAC type (*p* = 0.036). The overall inappropriate dosing rate was 30.8%, mainly driven by inappropriate underdosing (26.9%). These findings are consistent with previous Asian cohort studies, including a Korean nationwide study reporting frequent underdosing and lower label adherence, a Chinese atrial fibrillation cohort with an inappropriate dosing rate of 22.5%, and Japanese registry data reporting inappropriate dosing in approximately 20–30% of patients [16,17]. In the present cohort, edoxaban showed the highest appropriate dosing rate (73.3%), followed by apixaban (70.5%) and rivaroxaban (45.5%).

Several clinical factors may explain these prescribing patterns. Consistent with its lower renal clearance, apixaban was numerically prescribed more often in patients with lower eGFR [4]. In contrast, despite having the lowest prevalence of renal impairment, the rivaroxaban group showed a disproportionately high rate of off-label underdosing among patients who did not meet dose-reduction criteria. This pattern suggests empiric dose modification based on perceived bleeding risk rather than formal label criteria. Because once-daily rivaroxaban dosing may produce higher peak concentrations than twice-daily regimens, clinicians may be more concerned about bleeding risk in older or frail patients with borderline renal function [18]. However, the clinical benefit of empiric dose reduction remains controversial. Off-label underdosing has been reported in 26.2–39.6% of patients in previous studies, and prior evidence suggests that empiric dose-reduction does not consistently reduce bleeding risk and may be associated with increased mortality or adverse outcomes in selected cohorts [16,19,20]. In this context, laboratory-based exposure assessment may help contextualize dosing decisions when clinicians are concerned about overexposure despite label-based dosing [10].

The observed discordance between clinic clock-time and estimated pharmacokinetic windows illustrates an important limitation of interpreting routine outpatient spot sample DOAC concentrations. Although we applied an assumption-based pharmacokinetic window derived from expected time-to-peak profiles, measured concentrations were widely dispersed across both clock-time and assumed pharmacokinetic windows. Because exact dosing times were unavailable, pharmacokinetic window categorization was intended solely as an exploratory visualization framework to contextualize concentration distributions rather than to infer true peak or trough drug exposure. Therefore, documentation of the exact “time of last dose” is essential when DOAC concentration testing is ordered. Without this information, an apparently high or low concentration may reflect sampling timing rather than true overexposure or underexposure.

In the absence of exact dosing time or validated reference ranges, clinicians and laboratories interpreting a measured DOAC concentration in practice should consider it alongside the broader clinical context—including the time of last intake and blood sampling, prescribed dose and dosing frequency, Cockcroft–Gault creatinine clearance, age, body weight, concomitant antiplatelet or interacting medications, suspected non-adherence, and the specific clinical indication for testing—rather than in isolation. This contextual approach may be particularly relevant in scenarios such as urgent surgery, active bleeding, thrombosis occurring on therapy, deteriorating renal function, extremes of body weight, or empiric off-label dose-reduction. Preanalytical factors-missed doses, non-adherence, food intake, exact timing of the last dose, dynamic renal function, and interacting medications-likely also contributed to the observed variability but could not be captured in this retrospective dataset. This may be especially relevant for rivaroxaban, whose once-daily, food-dependent absorption can produce a more pronounced peak concentration than twice-daily regimens.

Routine coagulation assays showed variable associations with DOAC concentrations. In the present study, PT/INR demonstrated the strongest correlation with edoxaban concentrations (rs = 0.83, *p* < 0.001), followed by rivaroxaban (rs = 0.65) and apixaban (rs = 0.44). PT in seconds showed comparable correlations, whereas aPTT correlations were weaker and more variable across agents. Because INR was developed and validated for vitamin K antagonist monitoring, its applicability to DOAC exposure is not standardized; we therefore also report PT in seconds throughout, which yielded materially similar correlation coefficients to INR in our cohort. These findings are broadly consistent with previous studies reporting that PT may show moderate responsiveness to some direct factor Xa inhibitors, while aPTT generally has limited sensitivity for these agents [21,22]. Although routine PT/INR and aPTT may provide qualitative information regarding DOAC exposure in selected clinical contexts, they should not be interpreted as calibrated quantitative monitoring tools. Chromogenic anti-factor Xa assays therefore remain necessary when precise measurement of direct factor Xa inhibitor exposure is required.

The analytical comparison across platforms demonstrated excellent agreement between the CS-5100 and CN-6000 analyzers. Correlations were high with DiXaI reagents (apixaban, r = 0.885; edoxaban, r = 0.990; rivaroxaban, r = 0.918) and were consistently higher with LRT reagents (r = 0.993–0.996 across DOACs). Bland–Altman analysis also showed minimal systematic bias between analyzers, with mean biases of −1.86 ng/mL for apixaban, −6.86 ng/mL for edoxaban, and −10.99 ng/mL for rivaroxaban. Within-instrument comparisons between DiXaI and LRT reagents showed strong correlations, and 94.7–99.0% of measurements were within the limits of agreement. These findings suggest that platform-related differences are limited under the tested conditions, particularly when LRT reagents and drug-specific calibrators are used. Nevertheless, laboratory-specific verification remains necessary before applying measured concentrations to clinical interpretation.

The expected concentration ranges from outpatient spot samples should be interpreted as real-world distributions rather than therapeutic ranges. Lower concentration limits were relatively consistent across DOACs and assay methods, whereas upper limits were more variable, particularly for rivaroxaban. The CS-5100 DiXaI method yielded a higher and more variable upper limit for rivaroxaban (750.9 ng/mL; 90% CI, 376.8–898.6) than LRT-based methods and the CN-6000 platform (450.4–458.6 ng/mL), likely reflecting outlier influence in the smaller rivaroxaban cohort. In contrast, LRT-based methods produced narrower and more internally consistent real-world distributions across analyzers. These data are presented descriptively to characterize real-world exposure and should not be considered validated reference or therapeutic intervals.

Post-hoc descriptive analyses further illustrated the heterogeneity of real-world DOAC management. Among the 63 underdosed patients, 20 (31.7%) transitioned to guideline-appropriate regimens through dose escalation or agent switching, whereas 28 (44.4%) remained inappropriately underdosed. One fatal thromboembolic event occurred in the underdosing group in a 91-year-old patient with a high CHA_2_DS_2_-VASc score. However, given the approximately two-year interval between the index measurement and the event, as well as multiple comorbidities, no causal relationship can be inferred. Similarly, elevated concentration outliers occurred in both appropriately and inappropriately dosed patients and were not clearly associated with severe renal impairment or adverse clinical events. These findings suggest that plasma DOAC exposure cannot be inferred from dose alone and that selective laboratory testing may help contextualize dosing decisions in complex clinical situations [23,24].

Several limitations warrant consideration. First, the exact time of last DOAC administration was unavailable in this retrospective outpatient dataset; consequently, measured concentrations represent heterogeneous post-dose spot samples rather than protocolized peak/trough levels, and the pharmacokinetic-window categorization served solely as an exploratory visualization framework, not a validated pharmacokinetic reference. Second, the rivaroxaban sample size was relatively small (*n* = 38 samples/29 patients), yielding wider confidence intervals; these estimates should be regarded as exploratory and hypothesis-generating, sensitive to individual outliers, and require multicenter replication. Additionally, concentrations below the LoQ were excluded from quantitative analyses, which truncates the lower tail of the observed distribution; consequently, the reported 2.5th percentile limits in Table 4 likely represent a conservative (upper-bound) estimate rather than the true lower boundary of the underlying distribution. Third, dose appropriateness was determined using Cockcroft-Gault creatinine clearance per approved labeling, but discrepancies with other renal function estimates may have influenced classification in some patients; we also did not systematically capture CYP3A4/P-glycoprotein-modulating co-medications (e.g., amiodarone, verapamil, azole antifungals); consequently, edoxaban dose-reduction classification in this study was based on renal function and body weight only, and may not fully reflect all label-specified criteria, representing an unmeasured source of inter-individual variability. Fourth, because this cross-sectional design did not link concentrations to prospectively adjudicated bleeding, stroke, or mortality endpoints, no inference regarding the clinical utility of concentration monitoring can be drawn. Fifth, 25.7% of patients contributed multiple samples on separate visits; primary analyses were restricted to the first sample per patient. Sixth, correlation coefficients and regression slopes alone do not establish clinical interchangeability between platforms; although predefined acceptance criteria (mean bias within ±10%; ≥95% of differences within ±1.96 SD limits of agreement) were met under single-center conditions, multicenter validation across lot numbers and calibrator batches remains necessary. Finally, as a single-center study, generalizability may be limited.

## 5. Conclusions

We identified substantial heterogeneity in real-world DOAC dosing, frequently driven by off-label underdosing. The discordance between clinic clock-time and assumed pharmacokinetic windows highlights the importance of documenting the exact time of last dose when interpreting outpatient spot sample concentrations. Although routine PT/INR testing may provide qualitative information regarding DOAC exposure, chromogenic anti-factor Xa assays demonstrated excellent comparability between the CS-5100 and CN-6000 analyzers, particularly with LRT reagents; these findings are consistent with limited platform-related differences under the tested conditions, though interchangeability claims require multicenter validation and laboratory-specific verification. These findings support the analytical reliability of chromogenic anti-factor Xa assays for laboratory assessment of direct factor Xa inhibitor exposure. Because clinical outcomes were not systematically evaluated in this study, any implications for individualized anticoagulation management remain hypothesis-generating and require prospective validation.

## Figures and Tables

**Table 1:** Baseline characteristics of the study cohort (first sample per unique patient, *n* = 292).

Characteristic	Apixaban (*n* = 154)	Edoxaban (*n* = 109)	Rivaroxaban (*n* = 29)	*p*-Value *
Age, years, median (IQR)	73.0 (65.0–79.0)	73.0 (65.0–82.0)	73.0 (66.0–79.0)	0.670
Male sex, *n* (%)	97 (63.0)	66 (60.6)	22 (75.9)	0.312
Weight, kg, median (IQR)	66.3 (56.5–73.1)	63.0 (55.8–71.8)	66.5 (53.6–77.1)	0.628
Height, cm, median (IQR)	165.9 (158.8–170.3)	162.8 (155.0–169.0)	166.0 (157.2–169.9)	0.093
BMI, kg/m^2^, median (IQR)	24.2 (22.2–25.9)	23.8 (21.7–26.4)	24.4 (21.1–27.2)	0.926
Serum creatinine, mg/dL, median (IQR)	1.0 (0.8–1.2)	1.0 (0.8–1.2)	0.9 (0.8–1.0)	0.188
eGFR (CKD-EPI), mL/min/1.73 m^2^, median (IQR)	72.0 (57.0–85.0)	72.0 (55.0–87.0)	83.0 (67.0–88.0)	0.074
Cockcroft–Gault CrCl, mL/min, median (IQR)	59.2 (45.4–74.1)	56.8 (44.4–73.9)	68.2 (48.5–75.1)	0.580
CHA_2_DS_2_-VASc score, median (IQR)	3.0 (2.0–4.0)	3.0 (2.0–4.0)	4.0 (3.0–4.0)	0.745
HAS-BLED score, median (IQR)	1.0 (1.0–2.0)	1.0 (1.0–2.0)	1.0 (1.0–1.0)	0.240
Concomitant antiplatelet use, *n* (%)	32 (20.8)	35 (32.1)	4 (13.8)	0.041
Concomitant NSAID use, *n* (%)	2 (1.3)	1 (0.9)	1 (3.4)	0.577
Concomitant SSRI use, *n* (%)	1 (0.6)	2 (1.8)	0 (0.0)	0.544

Abbreviations: BMI, body mass index; CrCl, creatinine clearance; eGFR, estimated glomerular filtration rate; IQR, interquartile range; NSAID, nonsteroidal anti-inflammatory drug; SSRI, selective serotonin reuptake inhibitor. * Kruskal–Wallis test for continuous variables and chi-square test (or Fisher’s exact test where any expected cell count <5) for categorical variables.

**Table 2:** Assessment of dose appropriateness according to DOAC-specific dose-reduction criteria (first sample per unique patient, *n* = 234).

Characteristic	Apixaban (*n* = 122)	Edoxaban (*n* = 90)	Rivaroxaban (*n* = 22)	*p*-Value *
Appropriate dosing, *n* (%)	86 (70.5)	66 (73.3)	10 (45.5)	0.036
Inappropriate underdosing, *n* (%)	34 (27.9)	20 (22.2)	9 (40.9)	0.197
Inappropriate overdosing, *n* (%)	2 (1.6)	4 (4.4)	3 (13.6)	0.025
Meets dose-reduction criteria, *n* (%)	17 (13.9)	31 (34.4)	7 (31.8)	0.001
Received the recommended reduced dose among those meeting criteria, *n* (%)	13 (76.5)	25 (80.6)	3 (42.9)	0.114

Data are presented as *n* (%). Dose appropriateness and dose-reduction criteria were determined according to Food and Drug Administration (FDA)-approved labeling. Creatinine clearance calculated using the Cockcroft–Gault equation (CrCl_CG) was used for dose appropriateness classification. Apixaban dose-reduction was defined as 2.5 mg twice daily when at least two of the following criteria were met: age ≥ 80 years, body weight ≤ 60 kg, or serum creatinine ≥ 1.5 mg/dL. Edoxaban dose-reduction was defined as 30 mg once daily for CrCl_CG 15–50 mL/min or body weight ≤ 60 kg, and rivaroxaban dose-reduction as 15 mg once daily for CrCl_CG 15–50 mL/min. * *p* values were calculated using χ^2^ tests. Bonferroni correction was applied for pairwise comparisons (corrected α = 0.0167). Abbreviations: BID, twice daily; CrCl_CG, Cockcroft–Gault creatinine clearance; DOAC, direct oral anticoagulant; FDA, Food and Drug Administration; QD, once daily; SCr, serum creatinine.

**Table 3:** Inter-analyzer and inter-reagent method comparison of DOAC assays using Passing-Bablok regression. (**A**) CS-5100 vs. CN-6000 inter-analyzer comparison; (**B**) DiXaI vs. LRT within-analyzer inter-reagent comparison.

(**A**)						
**DOAC Type**	**Reagent**	***n***	**r**	**Slope (95% CI)**	**Intercept (95% CI)**	**Mean Bias** **(95% LoA)**
Apixaban	DiXaI	207	0.885	0.98 (0.92–1.05)	3.4 (−4.1 to 11.6)	−2.1 (−42.5 to 38.3)
	LRT		0.993	1.01 (0.99–1.02)	−1.2 (−3.5 to 1.4)	−0.8 (−18.4 to 16.8)
Edoxaban	DiXaI	146	0.990	1.02 (0.99–1.04)	−2.8 (−6.2 to 0.9)	−3.1 (−22.6 to 16.4)
	LRT		0.996	1.037 (1.014–1.059) †	−4.212 (−6.956 to −1.822) †	−4.6 (−14.8 to 5.6)
Rivaroxaban	DiXaI	38	0.918	0.97 (0.87–1.08)	8.4 (−12.3 to 28.6)	−3.5 (−98.4 to 91.4)
	LRT		0.994	1.02 (0.98–1.05)	−2.4 (−9.8 to 5.2)	−1.8 (−30.2 to 26.6)
(**B**)						
**DOAC Type**	**Analyzer**	***n***	**r**	**Slope (95% CI)**	**Intercept (95% CI)**	**Mean Bias** **(95% LoA)**
Apixaban	CS-5100	207	0.912	1.03 (0.98–1.08)	−4.1 (−9.3 to 1.4)	−2.8 (−38.4 to 32.8)
	CN-6000		0.968	1.01 (0.98–1.04)	−3.2 (−6.5 to 0.4)	−3.6 (−24.2 to 17.0)
Edoxaban	CS-5100	147 *	0.972	1.04 (1.00–1.08)	−3.8 (−7.5 to −0.4) †	−3.2 (−19.6 to 13.2)
	CN-6000	146	0.988	1.05 (1.02–1.08) †	−5.6 (−8.2 to −3.1) †	−4.8 (−15.4 to 5.8)
Rivaroxaban	CS-5100	38	0.895	1.02 (0.92–1.13)	−6.2 (−25.4 to 12.8)	−4.2 (−104.6 to 96.2)
	CN-6000		0.986	1.05 (1.01–1.10) †	−8.4 (−15.6 to −1.8) †	−6.1 (−32.4 to 20.2)

Abbreviations: CI, confidence interval; DiXaI, direct factor Xa inhibitor assay (BIOPHEN™); LoA, limits of agreement; LRT, liquid reagent technology (BIOPHEN™ Heparin LRT); r, correlation coefficient. Predefined analytical acceptance criteria: mean bias within ±10% and ≥95% of paired differences within ±1.96 SD limits of agreement. * Edoxaban was measured in 147 samples for all analyses using CS-5100, but one edoxaban sample yielded an invalid reading on CN-6000 on repeat testing and was excluded from all analyses using CN-6000 data, regardless of reagent (*n* = 146 for those specific comparisons). This exclusion applied uniformly to CN-6000 DiXaI, CN-6000 LRT, the CS-5100-vs.-CN-6000 inter-analyzer comparisons, and the CN-6000 LRT-based PT/aPTT correlation analyses (Figure 3). † Slope 95% CI does not include 1.0, or intercept 95% CI does not include 0, indicating statistically significant proportional or constant bias. These findings indicate that strong correlation does not by itself establish clinical interchangeability; multicenter validation across different lot numbers and calibrator batches remains necessary. Rivaroxaban estimates (*n* = 38 samples/29 unique patients) are exploratory and sensitive to individual outliers.

**Table 4:** Observed plasma concentration distributions of direct oral factor Xa inhibitors in outpatient spot samples.

DOAC Type	Instrument and Method	2.5% Limit (90% CI) (ng/mL)	97.5% Limit (90% CI) (ng/mL)
Apixaban	CS-5100 (DiXaI)	35.0 (31.3 to 41.8)	449.5 (318.8 to 593.2)
	CS-5100 (LRT)	34.3 (30.5 to 41.3)	394.4 (301.3 to 530.2)
	CN-6000 (DiXaI)	37.9 (31.7 to 40.6)	450.6 (303.1 to 589.5)
	CN-6000 (LRT)	36.5 (33.4 to 42.6)	410.7 (305.2 to 556.2)
Edoxaban	CS-5100 (DiXaI)	32.9 (30.8 to 36.1)	454.8 (396.3 to 986.6)
	CS-5100 (LRT)	33.9 (23.7 to 36.0)	424.8 (384.2 to 579.5)
	CN-6000 (DiXaI)	30.1 (29.4 to 34.4)	433.3 (358.8 to 469.3)
	CN-6000 (LRT)	31.1 (30.1 to 32.9)	408.8 (371.7 to 464.3)
Rivaroxaban	CS-5100 (DiXaI)	35.3 (32.9 to 54.1)	750.9 (376.8 to 898.6)
	CS-5100 (LRT)	37.8 (34.6 to 47.2)	451.0 (400.0 to 457.4)
	CN-6000 (DiXaI)	35.8 (31.1 to 50.7)	458.6 (399.3 to 472.8)
	CN-6000 (LRT)	38.5 (35.0 to 47.9)	450.4 (388.2 to 463.8)

These values represent the central 95% distribution of outpatient post-dose spot sample concentrations, derived from heterogeneous dosing regimens and sampling times, and must not be interpreted as validated reference or therapeutic ranges. The 2.5th and 97.5th percentile limits for apixaban and edoxaban were calculated using non-parametric quantile estimation (CLSI EP28-A3c); rivaroxaban limits were calculated using the Harrell–Davis bootstrap method due to the smaller sample size. The CS-5100 DiXaI method for rivaroxaban showed a notably higher and more variable upper limit, reflecting the influence of outliers in the small sample. LRT-based methods and CN-6000 methods showed more consistent upper limits (450.4–458.6 ng/mL). The 90% confidence intervals are provided for both lower and upper percentile limits. Abbreviations: CI, confidence interval; CLSI, Clinical and Laboratory Standards Institute; LRT, liquid reagent technology.

## Data Availability

The data presented in this study are available from the corresponding author upon reasonable request. The data are not publicly available due to institutional and privacy restrictions.

## Supplementary Materials

The following supporting information can be downloaded at: https://www.mdpi.com/article/10.3390/diagnostics16172725/s1, Supplementary Methods S1: Assumption-based pharmacokinetic-window classification; Supplementary Methods S2: Post hoc review and exposure outlier analysis; Table S1: Outpatient clock-time window distribution of plasma DOAC concentrations; Table S2: Correlation between DOAC levels determined by CN-6000 LRT and other parameters; Table S3: Comparison of LRT and DiXaI reagents across analyzers; Figure S1: Assumption-based pharmacokinetic window classification; Figure S2: Bland–Altman plots for CS-5100 versus CN-6000; Figure S3: Bland–Altman plots comparing LRT and DiXaI reagents. References [25,26,27,28,29,30,31] are cited in the supplementary materials.
