# Supplementary material for: Real-World Assessment of Direct Oral Factor Xa Inhibitors: Dosing Appropriateness, Drug Exposure, and Inter-Platform Comparability of Chromogenic Anti-Xa Assays
Source: Diagnostics (Basel). 2026 Aug 26;16(17):2725. doi: 10.3390/diagnostics16172725 (PMC13565395; doi:10.3390/diagnostics16172725)
Supplement: Supplementary file 1 [file diagnostics-16-02725-s001.zip › diagnostics-4390296-supplementary.pdf]

Supplementary Methods S1. Assumption-based pharmacokinetic-window classification

The exact time of direct oral anticoagulant (DOAC) administration was not recorded in this retrospective outpatient dataset. For exploratory visualization only, we set a nominal 07:00 dose time and calculated the elapsed time from that nominal time to the blood-collection time. Each sample was then grouped into four descriptive elapsed-time windows (1–4 h, 4–8 h, 8–12 h, >12 h). We emphasize that these windows are not validated pharmacokinetic peak, intermediate, or trough windows: adherence to a morning-dose instruction was not objectively verified, dosing schedules differ between drugs, and patients may not have taken their medication at the nominal time. The window categorization is therefore intended solely as an exploratory descriptive framework and must not be used to infer individual pharmacokinetic exposure. Any results derived from this categorization (including Supplementary Figure S1) should be interpreted as hypothesis-generating only.

The peak window (1–4 h) was pre-selected to encompass the time of maximum plasma concentration (Cmax) reported on the FDA label for the three assessed factor Xa inhibitors. Specifically, the US prescribing information for apixaban (Eliquis) states Cmax occurs 3–4 h after oral administration. For edoxaban (SAVAYSA), the Food and Drug Administration (FDA) label describes the peak effect as occurring within 1–2 h after oral administration, which aligns with the time to maximum concentration for edoxaban. For rivaroxaban (XARELTO), the FDA label states that the Cmax is reached 2–4 h after tablet ingestion. Therefore, for all DOAC, the time to peak concentration was defined to align with the absorption/peak concentration timeframe on the FDA-approved label and existing literature (1 to 4 h post-administration) [25-27].

Supplementary Methods S2. Post-hoc review and exposure outlier analysis

Electronic medical records were retrospectively reviewed to assess subsequent dose modifications, treatment discontinuation, loss to follow-up, and clinical events among patients with inappropriate DOAC dosing. The primary dose-appropriateness analysis used the first available visit per patient, whereas the longitudinal review included patients with inappropriate dosing identified at any available visit. Underdosing and overdosing subgroups were mutually exclusive. Major bleeding was defined according to the International Society on Thrombosis and Haemostasis (ISTH) criteria, and clinically relevant non-major bleeding followed ISTH definitions [28,29]. Thromboembolic events included ischemic stroke, transient ischemic attack, and systemic embolism in accordance with the 2021 European Heart Rhythm Association Practical Guide on the Use of Non-Vitamin K Antagonist Oral Anticoagulant and 2023 American College of Cardiology/American Heart Association Atrial fibrillation guidelines [30,31]. Outcomes were summarized as crude event counts.

References

[1] Tran HA, Merriman E, Baker R, Curnow J, Young L, Tan CW, et al. 2025 Guidelines for direct oral anticoagulants: a practical guidance on the prescription, laboratory testing, peri-operative and bleeding management. Intern Med J 2025;55(7):1174-83. https://doi.org/10.1111/imj.70103.

[2] Connors JM. Testing and monitoring direct oral anticoagulants. Blood 2018;132(19):2009-15. https://doi.org/10.1182/blood-2018-04-791541.

[3] Ment J. Direct oral anticoagulants: key considerations for use to prevent stroke in patients with nonvalvular atrial fibrillation. Vasc Health Risk Manag 2015;11:317-32. https://doi.org/10.2147/vhrm.S79065.

[4] Hindley B, Lip GYH, McCloskey AP, Penson PE. Pharmacokinetics and pharmacodynamics of direct oral anticoagulants. Expert Opin Drug Metab Toxicol 2023;19(12):911-23. https://doi.org/10.1080/17425255.2023.2287472.

[5] Rienstra M, Tzeis S, Bunting KV, Caso V, Crijns H, De Potter TJR, et al. Spotlight on the 2024 ESC/EACTS management of atrial fibrillation guidelines: 10 novel key aspects. Europace 2024;26(12). https://doi.org/10.1093/europace/euae298.

[6] Hörber S, Lehmann R, Peter A. Evaluation of the Atellica COAG 360 coagulation analyzer in a central laboratory of a maximum care hospital. Int J Lab Hematol 2020;42(1):28-36. https://doi.org/10.1111/ijlh.13119.

[7] Königsbrügge O, Quehenberger P, Belik S, Weigel G, Seger C, Griesmacher A, et al. Anti-coagulation assessment with prothrombin time and anti-Xa assays in real-world patients on treatment with rivaroxaban. Ann Hematol 2015;94(9):1463-71. https://doi.org/10.1007/s00277-015-2407-y.

[8] Schmitz EM, Boonen K, van den Heuvel DJ, van Dongen JL, Schellings MW, Emmen JM, et al. Determination of dabigatran, rivaroxaban and apixaban by ultra-performance liquid chromatography - tandem mass spectrometry (UPLC-MS/MS) and coagulation assays for therapy monitoring of novel direct oral anticoagulants. J Thromb Haemost 2014;12(10):1636-46. https://doi.org/10.1111/jth.12702.

[9] Douxfils J, Adcock DM, Bates SM, Favaloro EJ, Gouin-Thibault I, Guillermo C, et al. 2021 Update of the International Council for Standardization in Haematology Recommendations for Laboratory Measurement of Direct Oral Anticoagulants. Thromb Haemost 2021;121(8):1008-20. https://doi.org/10.1055/a-1450-8178.

[10] Gosselin RC, Adcock DM, Bates SM, Douxfils J, Favaloro EJ, Gouin-Thibault I, et al. International Council for Standardization in Haematology (ICSH) Recommendations for Laboratory Measurement of Direct Oral Anticoagulants. Thromb Haemost 2018;118(3):437-50. https://doi.org/10.1055/s-0038-1627480.

[11] Gosselin RC, Cuker A. Assessing Direct Oral Anticoagulants in the Clinical Laboratory. Clin Lab Med 2024;44(3):551-62. https://doi.org/10.1016/j.cll.2024.04.014.

[12] Granger CB, Alexander JH, McMurray JJ, Lopes RD, Hylek EM, Hanna M, et al. Apixaban versus warfarin in patients with atrial fibrillation. N Engl J Med 2011;365(11):981-92. https://doi.org/10.1056/NEJMoa1107039.

[13] Giugliano RP, Ruff CT, Braunwald E, Murphy SA, Wiviott SD, Halperin JL, et al. Edoxaban versus warfarin in patients with atrial fibrillation. N Engl J Med 2013;369(22):2093-104. https://doi.org/10.1056/NEJMoa1310907.

[14] Kim JY, Choi EK, Lim HE, Oh YS, Cho Y, On YK. Outcomes of On-Label Reduced-Dose Edoxaban in Patients With Atrial Fibrillation: The LEDIOS Registry. J Korean Med Sci 2022;37(48):e335. https://doi.org/10.3346/jkms.2022.37.e335.

[15] Michels WM, Grootendorst DC, Verduijn M, Elliott EG, Dekker FW, Krediet RT. Performance of the Cockcroft-Gault, MDRD, and new CKD-EPI formulas in relation to GFR, age, and body size. Clin J Am Soc Nephrol 2010;5(6):1003-9. https://doi.org/10.2215/cjn.06870909.

[16] Yu HT, Yang PS, Jang E, Kim TH, Uhm JS, Kim JY, et al. Label Adherence of Direct Oral Anticoagulants Dosing and Clinical Outcomes in Patients With Atrial Fibrillation. J Am Heart Assoc 2020;9(12):e014177. https://doi.org/10.1161/jaha.119.014177.

[17] Guo X, Du J, Yang Y, Wu M, Ou W, Han X, et al. Edoxaban for stroke prevention in atrial fibrillation and factors associated with dosing: patient characteristics from the prospective observational ETNA-AF-China registry. Sci Rep 2024;14(1):2778. https://doi.org/10.1038/s41598-024-51776-3.

[18] Almalbis CA, Md Redzuan A, Andrada CP, Gonzaga NA, Mohd Saffian S. Peak and trough concentrations of apixaban and rivaroxaban in adult patients: a systematic review and meta-analysis. J Thromb Haemost 2025;23(4):1289-314. https://doi.org/10.1016/j.jtha.2024.12.032.

[19] Maura G, Pariente A, Alla F, Billionnet C. Adherence with direct oral anticoagulants in nonvalvular atrial fibrillation new users and associated factors: a French nationwide cohort study. Pharmacoepidemiol Drug Saf 2017;26(11):1367-77. https://doi.org/10.1002/pds.4268.

[20] Shen NN, Ferroni E, Amidei CB, Canova C, Peron V, Wang JL, et al. An Updated Pooled Analysis of Off-Label Under and Over-Dosed Direct Oral Anticoagulants in Patients with Atrial Fibrillation. Clin Appl Thromb Hemost 2023;29:10760296231179439. https://doi.org/10.1177/10760296231179439.

[21] Samuelson BT, Cuker A, Siegal DM, Crowther M, Garcia DA. Laboratory Assessment of the Anticoagulant Activity of Direct Oral Anticoagulants: A Systematic Review. Chest 2017;151(1):127-38. https://doi.org/10.1016/j.chest.2016.08.1462.

[22] Toorop MMA, van Rein N, Nierman MC, Vermaas HW, Huisman MV, van der Meer FJM, et al. Inter- and intra-individual concentrations of direct oral anticoagulants: The KIDOAC study. J Thromb Haemost 2022;20(1):92-103. https://doi.org/10.1111/jth.15563.

[23] Camm AJ, Cools F, Virdone S, Bassand JP, Fitzmaurice DA, Arthur Fox KA, et al. Mortality in Patients With Atrial Fibrillation Receiving Nonrecommended Doses of Direct Oral Anticoagulants. J Am Coll Cardiol 2020;76(12):1425-36. https://doi.org/10.1016/j.jacc.2020.07.045.

[24] Liu X, Huang M, Ye C, Xiao X, Yan C. Effect of non-recommended doses versus recommended doses of direct oral anticoagulants in atrial fibrillation patients: A meta-analysis. Clin Cardiol 2021;44(4):472-80. https://doi.org/10.1002/clc.23586.

[25] Ogata K, Mendell-Harary J, Tachibana M, Masumoto H, Oguma T, Kojima M, et al. Clinical safety, tolerability, pharmacokinetics, and pharmacodynamics of the novel factor Xa inhibitor edoxaban in healthy volunteers. J Clin Pharmacol 2010;50(7):743-53. https://doi.org/10.1177/0091270009351883.

[26] Frost C, Wang J, Nepal S, Schuster A, Barrett YC, Mosqueda-Garcia R, et al. Apixaban, an oral, direct factor Xa inhibitor: single dose safety, pharmacokinetics, pharmacodynamics and food effect in healthy subjects. Br J Clin Pharmacol 2013;75(2):476-87. https://doi.org/10.1111/j.1365-2125.2012.04369.x.

[27] Mueck W, Stampfuss J, Kubitza D, Becka M. Clinical pharmacokinetic and pharmacodynamic profile of rivaroxaban. Clin Pharmacokinet 2014;53(1):1-16. https://doi.org/10.1007/s40262-013-0100-7.
[28] Schulman S, Kearon C. Definition of major bleeding in clinical investigations of antihemostatic medicinal products in non-surgical patients. J Thromb Haemost 2005;3(4):692-4. https://doi.org/10.1111/j.1538-7836.2005.01204.x.
[29] Kaatz S, Ahmad D, Spyropoulos AC, Schulman S. Definition of clinically relevant non-major bleeding in studies of anticoagulants in atrial fibrillation and venous thromboembolic disease in non-surgical patients: communication from the SSC of the ISTH. J Thromb Haemost 2015;13(11):2119-26. https://doi.org/10.1111/jth.13140.
[30] Steffel J, Collins R, Antz M, Cornu P, Desteghe L, Haeusler KG, et al. 2021 European Heart Rhythm Association Practical Guide on the Use of Non-Vitamin K Antagonist Oral Anticoagulants in Patients with Atrial Fibrillation. Europace 2021;23(10):1612-76. https://doi.org/10.1093/europace/euab065.
[31] Joglar JA, Chung MK, Armbruster AL, Benjamin EJ, Chyou JY, Cronin EM, et al. 2023 ACC/AHA/ACCP/HRS Guideline for the Diagnosis and Management of Atrial Fibrillation: A Report of the American College of Cardiology/American Heart Association Joint Committee on Clinical Practice Guidelines. Circulation 2024;149(1):e1-e156. https://doi.org/10.1161/cir.0000000000001193.
