# Supplementary material for: Real-World Assessment of Direct Oral Factor Xa Inhibitors: Dosing Appropriateness, Drug Exposure, and Inter-Platform Comparability of Chromogenic Anti-Xa Assays
Source: Diagnostics (Basel). 2026 Aug 26;16(17):2725. doi: 10.3390/diagnostics16172725 (PMC13565395; doi:10.3390/diagnostics16172725)
Supplement: Supplementary file 1 [file diagnostics-16-02725-s001.zip › Supplemental_Data_r2.1.pdf]

Supplementary materials

Supplementary Figure S1. Assumption-based pharmacokinetic window classification of outpatient direct oral anticoagulant (DOAC) concentrations (assumed 07:00 dosing) with atrial fibrillation expected on-therapy ranges.

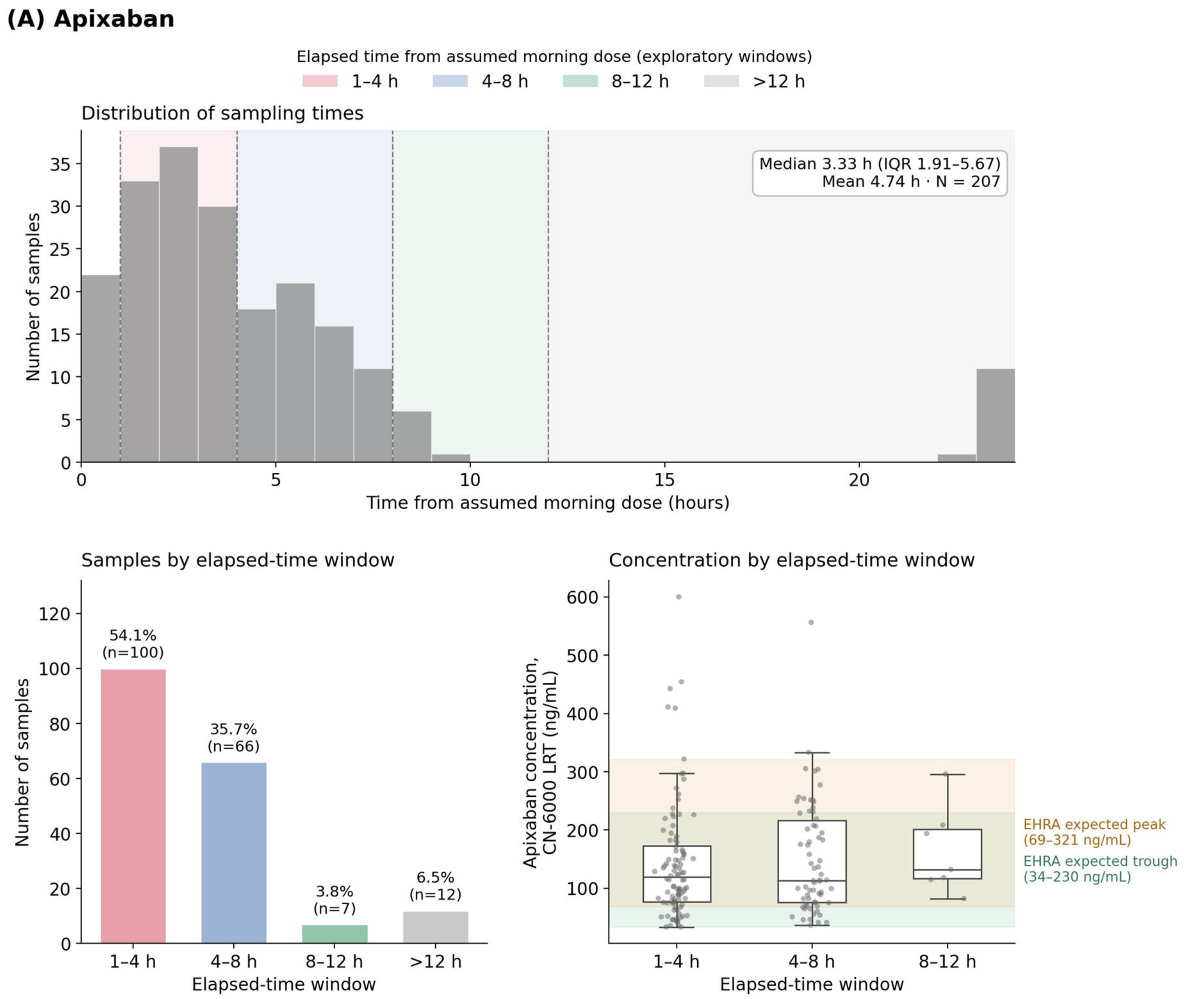

(B) Edoxaban

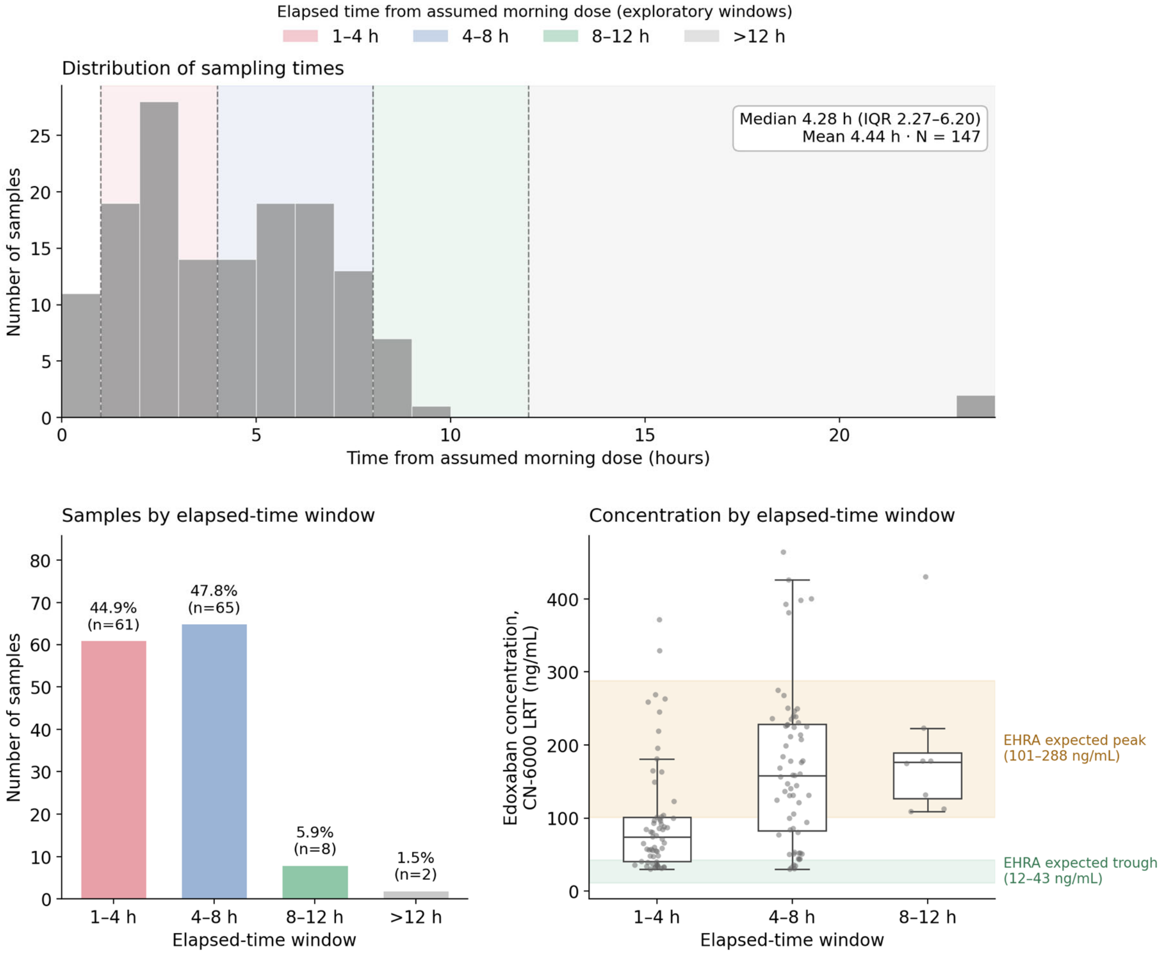
Elapsed time from assumed morning dose (exploratory windows)
1–4 h
4–8 h
8–12 h
>12 h
Distribution of sampling times
Median 4.28 h (IQR 2.27–6.20)
Mean 4.44 h · N = 147
Number of samples
Time from assumed morning dose (hours)
Samples by elapsed-time window
44.9% (n=61)
47.8% (n=65)
5.9% (n=8)
1.5% (n=2)
Elapsed-time window
Concentration by elapsed-time window
Edoxaban concentration, CN-6000 LRT (ng/mL)
EHRA expected peak (101–288 ng/mL)
EHRA expected trough (12–43 ng/mL)

(C) Rivaroxaban

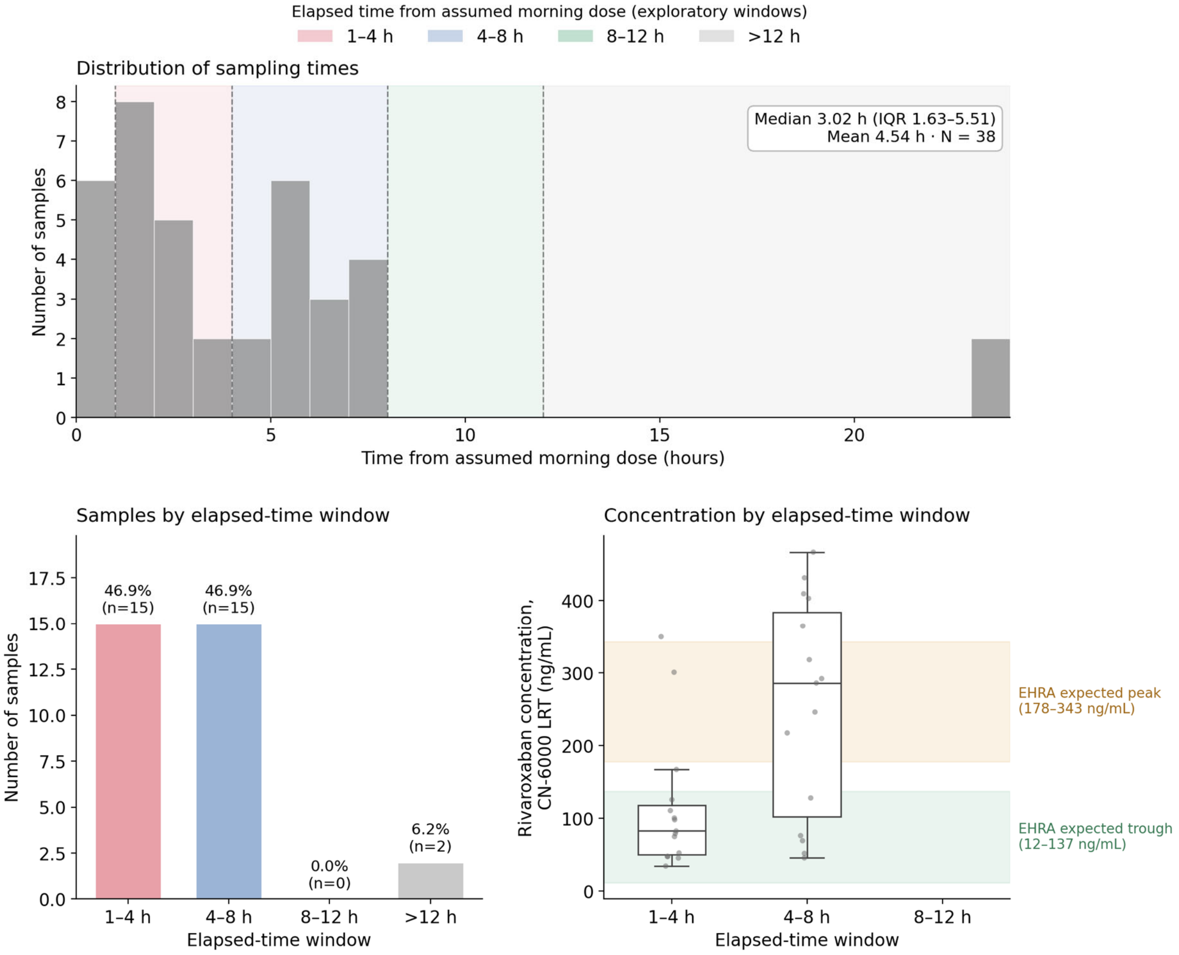

Outpatient blood-draw times were visualized and mapped to elapsed-time intervals assuming a nominal 07:00 dose. Samples of (A) apixaban, (B) edoxaban and (C) rivaroxaban were categorized into descriptive elapsed-time windows (1–4 h, 4–8 h, 8–12 h, >12 h) for exploratory visualization only. Because the exact time of last dose was not recorded, these windows do not represent true pharmacokinetic peak, intermediate, or trough exposures, and the shaded on-therapy bands are provided solely for descriptive comparison and must not be interpreted as validated pharmacokinetic reference intervals. Panels display concentration distributions across these assumed elapsed-time windows and/or clock-time distributions (format consistent across DOACs). Shaded bands indicate expected on-therapy trough and peak plasma concentration ranges for patients with atrial fibrillation (AF) from the European Heart Rhythm Association Practical Guide: apixaban trough 34–230 ng/mL and peak 69–321 ng/mL; edoxaban trough 12–43 ng/mL and peak 101–288 ng/mL; rivaroxaban trough 12–137 ng/mL and peak 178–343 ng/mL.

Supplementary Figure S2. Bland–Altman plots for CS-5100 versus CN-6000 analyzer comparison by direct oral anticoagulant (DOAC) type.

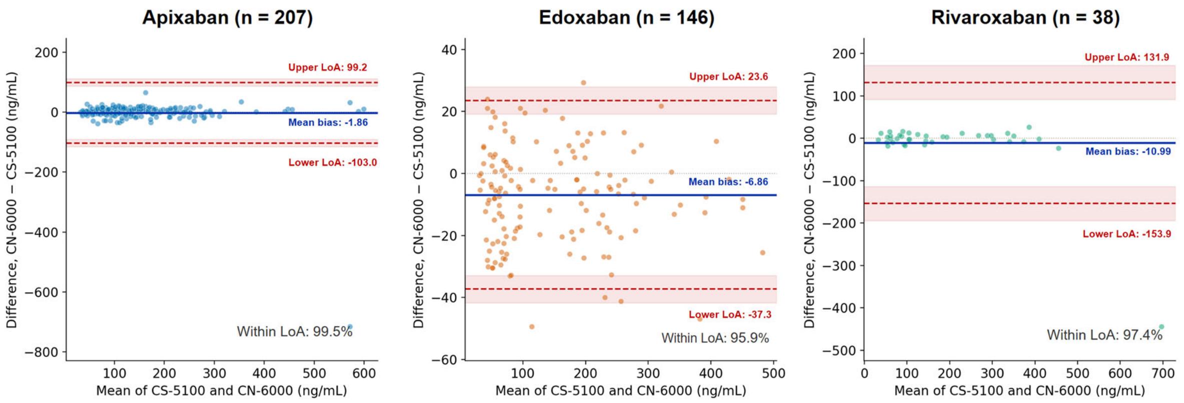

The x-axis represents the mean of the two methods, and the y-axis represents the difference (CN-6000 - CS-5100). The blue dashed line indicates the mean difference (bias), and red dashed lines indicate the limits of agreement (mean ± 1.96 SD). Shaded areas represent 95% confidence intervals for the limits of agreement. All three DOACs showed ≥95% of measurements within the limits of agreement, indicating strong analytical concordance under the tested single-center conditions. Correlation and limits-of-agreement metrics alone, however, do not establish clinical interchangeability; multicenter validation with predefined acceptance criteria (mean bias within ±10%; ≥95% of paired differences within ±1.96 SD limits of agreement) remains necessary.

Supplementary Figure S3. Bland–Altman plots assessing agreement between liquid reagent technology (LRT) and DiXaI reagents for direct oral anticoagulant concentration measurement.

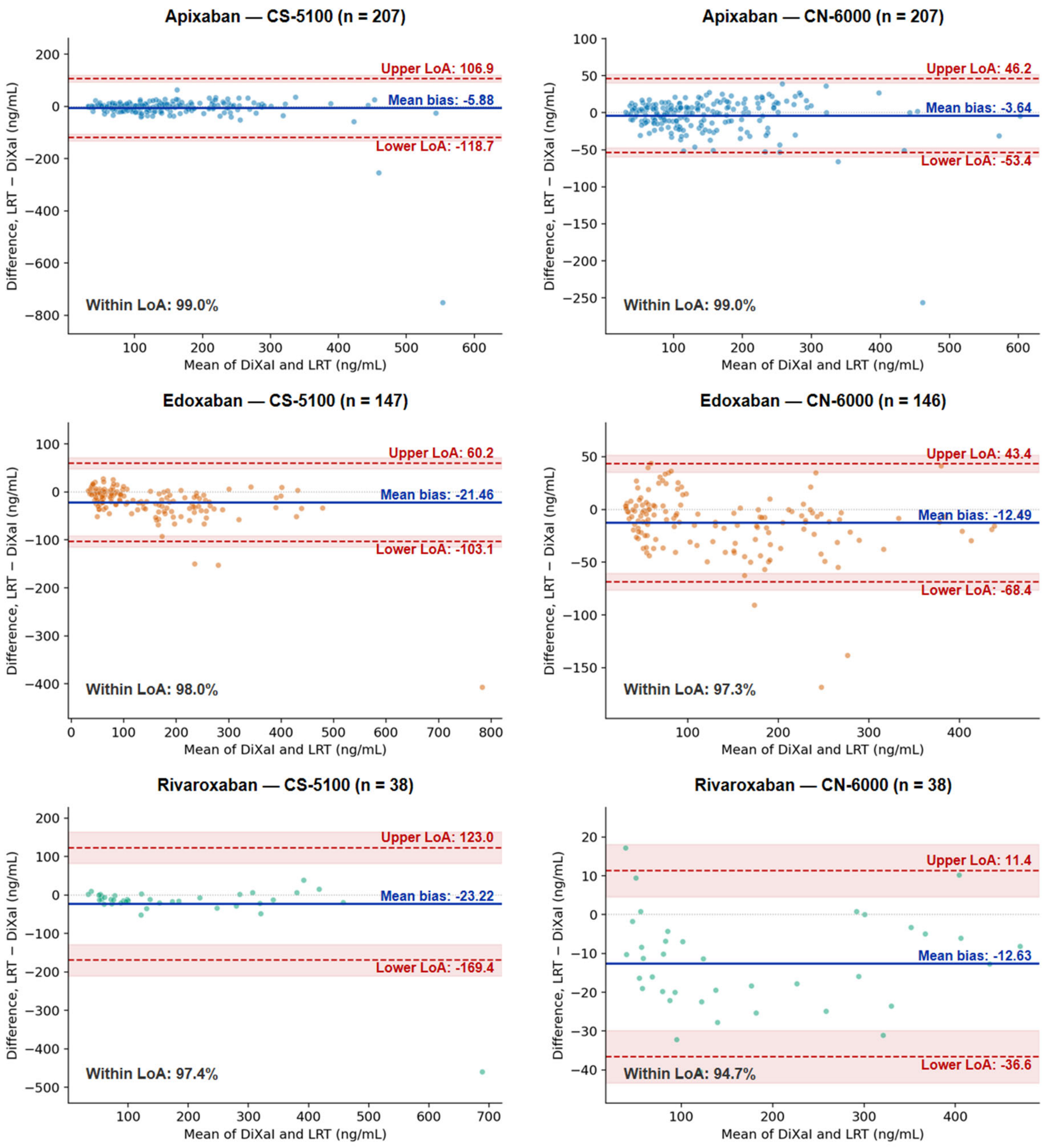

Bland–Altman plots show agreement between LRT and DiXaI reagent measurements for apixaban (top, n = 207 samples / 154 unique patients), edoxaban (middle, n = 146 samples for the CN-6000 DiXaI-vs-LRT comparison and n = 147 samples for the CS-5100 DiXaI-vs-LRT comparison owing to one invalid CN-6000 DiXaI reading on repeat testing; 109 unique patients), and rivaroxaban (bottom, n = 38 samples / 29 unique patients) using CS-5100 (left) and CN-6000 (right) analyzers. The x-axis represents the mean of both methods, and the y-

axis represents the difference (LRT − DiXaI). The blue dashed line indicates the mean difference (bias), red dashed lines indicate the limits of agreement (mean ± 1.96 SD), and the gray line indicates zero difference. Mean bias ranged from −3.64 to −23.22 ng/mL, with 94.7–99.0% of measurements within the limits of agreement, supporting good analytical agreement between LRT and DiXaI reagents under the tested conditions. Abbreviations: LRT, liquid reagent technology; SD, standard deviation.

Supplementary Table S1. Outpatient clock-time window distribution of plasma direct oral anticoagulant concentrations measured by chromogenic anti-factor Xa assay (CN-6000, Liquid Reagent Technology), stratified by direct oral anticoagulant (DOAC) type.

DOAC	Clock_window	n	%	Conc_median [IQR] (ng/mL)	Conc_range (ng/mL)
Apixaban (n=207)	<06	1	0.5	98.8 [-]	98.8
	06–10	103	49.8	124.0 [79.0–181.4]	31.0–454.3
	10–14	85	41.1	115.2 [75.2–201.7]	33.4–600.0
	14–18	18	8.7	116.5 [97.8–208.0]	41.3–295.9
	18–24	0	0		
Edoxaban (n=147)	<06	0	0		
	06–10	60	40.8	65.5 [40.9–101.3]	30.1–371.7
	10–14	66	44.9	144.3 [57.5–227.8]	30.1–464.3
	14–18	21	14.3	174.5 [112.3–183.8]	50.9–430.4
	18–24	0	0		
Rivaroxaban (n=38)	<06	0	0		
	06–10	21	55.3	82.8 [55.7–118.3]	34.5–349.7
	10–14	13	34.2	217.4 [69.4–292.0]	45.8–431.2
	14–18	4	10.5	363.6 [257.6–423.5]	76.3–466.5
	18–24	0	0		

Plasma DOAC concentrations are summarized by outpatient blood-draw clock-time windows (<06, 06–10, 10–14, 14–18, 18–24) for apixaban (n = 207 samples / 154 unique patients), edoxaban (n = 147 samples / 109 unique patients), and rivaroxaban (n = 38 samples / 29 unique patients). Concentrations are reported as median [IQR] and range (ng/mL). Percentages denote the proportion of samples within each clock-time window for the corresponding DOAC cohort. Concentrations shown here were quantified using a chromogenic anti-Xa–based method on the Sysmex CN-6000 platform with liquid reagent technology (LRT) reagents. Clock-time windows reflect outpatient workflow and do not necessarily correspond to pharmacokinetic peak/trough phases because the exact time since last dose was not standardized.

Abbreviations. DOAC, direct oral anticoagulant; IQR, interquartile range; LRT, Liquid Reagent Technology; anti-Xa, anti-factor Xa.

Supplementary Table S2. Correlation between direct oral anticoagulant (DOAC) levels determined by CN-6000 LRT and other parameters

DOAC	Parameter	Rs	CI_Lower	CI_Upper	P_value	DOAC_mean	DOAC_std	Param_mean	Param_std
Apixaban	Age (years)	0.144	0.008	0.275	0.0385	145.3	93.9	71.8	10.4
	Body weight (kg)	-0.08	-0.227	0.07	0.294	143.7	98.7	65.3	11.9
	eGFR (CKD-EPI) (mL/min/1.73m^2)	-0.127	-0.259	0.01	0.0682	145.3	93.9	65.7	22.9
	Serum creatinine (mg/dL)	0.061	-0.076	0.196	0.3816	145.3	93.9	1.2	1
	PT (INR)	0.436	0.318	0.54	<0.0001	145.3	93.9	1.1	0.1
	PT (seconds)	0.453	0.338	0.555	<0.0001	145.3	93.9	12.5	1.1
	aPTT (seconds)	0.153	0.01	0.289	0.0366	142.5	94.2	28	2.5
	CHA_2DS_2-VASc score	0.09	-0.047	0.223	0.1995	145.3	93.9	3.5	1.4
	HAS-BLED score	0.092	-0.045	0.225	0.1891	145.3	93.9	1.3	0.8
Edoxaban	Age (years)	-0.151	-0.306	0.012	0.0687	133.6	99.8	71.9	11
	Body weight (kg)	0.202	0.024	0.367	0.0267	131.9	99.6	64.3	11.8
	eGFR (CKD-EPI) (mL/min/1.73m^2)	0.06	-0.103	0.22	0.4711	133.6	99.8	69.3	20
	Serum creatinine (mg/dL)	-0.011	-0.173	0.152	0.8973	133.6	99.8	1	0.3
	PT (INR)	0.826	0.766	0.871	<0.0001	133.6	99.8	1.3	0.3
	PT (seconds)	0.819	0.758	0.866	<0.0001	133.6	99.8	14.2	3.4
	aPTT (seconds)	0.69	0.592	0.767	<0.0001	134.2	100.5	29.6	3.4
	CHA_2DS_2-VASc score	-0.084	-0.243	0.08	0.3148	133.6	99.8	3.2	1.5
	HAS-BLED score	-0.124	-0.281	0.039	0.1363	133.6	99.8	1.3	0.9
Rivaroxaban	Age (years)	-0.28	-0.551	0.043	0.0884	169.7	133.2	72	11.5
	Body weight (kg)	0.325	-0.04	0.613	0.0797	193.8	139.5	66.8	13.6
	eGFR (CKD-EPI) (mL/min/1.73m^2)	0.218	-0.109	0.503	0.1879	169.7	133.2	77.2	16.4
	Serum creatinine (mg/dL)	-0.012	-0.33	0.309	0.9444	169.7	133.2	0.9	0.3
	PT (INR)	0.651	0.418	0.804	<0.0001	169.7	133.2	1.3	0.2
	PT (seconds)	0.651	0.418	0.803	<0.0001	169.7	133.2	14.1	1.7
	aPTT (seconds)	0.386	0.03	0.655	0.0352	183.7	141.9	33.3	7
	CHA_2DS_2-VASc score	-0.134	-0.435	0.194	0.4236	169.7	133.2	3.6	1.5
	HAS-BLED score	-0.024	-0.341	0.298	0.8876	169.7	133.2	1	0.6

Correlations are based on CN-6000 LRT measurements. Unless otherwise noted, sample sizes represent the total number of samples in each group (Apixaban: 207 samples / 154 unique patients, Edoxaban: 147 samples / 109 unique patients [one sample yielded an invalid reading on CN-6000; n = 146 for analyses using CN-6000 data], Rivaroxaban: 38 samples / 29 unique patients). Because 75 patients (25.7%) contributed multiple samples on separate visits, results were consistent with the first-per-patient analysis. Data availability varied for body weight (n=173,121,30, respectively) and aPTT (n=188,143,30, respectively).

Abbreviations: Rs, Spearman's rank correlation coefficient; CI, confidence interval; mean, DOAC concentration; Param, Parameter value; eGFR, estimated glomerular filtration rate; PT, prothrombin time; INR, international normalized ratio; aPTT, activated partial thromboplastin time.

Supplementary Table S3. Comprehensive comparison of liquid reagent technology (LRT) and DiXaI reagent for direct oral anticoagulant (DOAC) measurements across CS-5100 and CN-6000 analyzers by DOAC type

DOAC Type	Instrument	Standard Mean (SD)	Standard CV%	LRT Mean (SD)	LRT CV%	Pearson r	Spearman rs	R²	Bias	LOA	Within LOA %
Apixaban (n=207)	CS-5100	150.8 (111.0)	73.6	145.0 (94.0)	64.8	0.855	0.972	0.731	-5.88	-118.7 to 106.9	99.0
	CN-6000	149.0 (98.0)	65.8	145.3 (93.9)	64.6	0.966	0.973	0.933	-3.64	-53.4 to 46.2	99.0
Edoxaban (n=146)	CS-5100	158.6 (127.4)	80.3	137.2 (104.6)	76.2	0.954	0.944	0.911	-21.46	-103.1 to 60.2	98.0
	CN-6000	146.1 (106.8)	73.1	133.6 (99.8)	74.7	0.964	0.921	0.930	-12.49	-68.4 to 43.4	97.3
Rivaroxaban (n=38)	CS-5100	193.3 (172.3)	89.1	170.1 (133.7)	78.6	0.911	0.988	0.831	-23.22	-169.4 to 123.0	97.4
	CN-6000	182.3 (132.5)	72.7	169.7 (133.2)	78.5	0.996	0.982	0.992	-12.63	-36.6 to 11.4	94.7

Passing–Bablok regression and Bland–Altman analysis were performed to assess agreement between LRT and DiXaI reagent measurements. Correlation coefficients represent the relationship between LRT and DiXaI reagent measurements for each DOAC-analyzer combination.

Abbreviations: DOAC, direct oral anticoagulant; LRT, liquid reagent technology; SD, standard deviation; CV, coefficient of variation; r, Pearson correlation coefficient; rs, Spearman correlation coefficient; R^2, coefficient of determination; LOA, 95% limits of agreement.

General note on interpretation. Throughout this Supplementary Data document, all reported concentration distributions and inter-analyzer/inter-reagent agreement metrics reflect outpatient post-dose spot samples of uncertain elapsed time from the last dose. They must not be interpreted as validated pharmacokinetic reference intervals, therapeutic ranges, or evidence of clinical interchangeability between analyzers. All rivaroxaban-specific estimates (n = 38 samples / 29 unique patients) are exploratory and sensitive to individual outliers.
